# Practical Departments

**Published:** 1903-05-09

**Authors:** 


### PRACTICAL DEPARTMENTS.

### BEDS FOR HOSPITALS AND INSTITUTIONS.

Messrs. Chorlton, of Manchester, have sent us their catalogue of beds and bedding. Amongst the comprehensive list we especially note some very excellent beds suitable for hospitals and nursing homes. Messrs. Chorlton have grasped the main features necessary in furniture for such purposes, namely, absence of ornaments or projections which can trap dust, solidity, and moderate price. Their "Excelsior" bed is one that meets most requirements, and to it can be attached a simple self-elevator or pulley. For surgical purposes there is a special "Surgical" bedstead, with removable head, and space between the tube sides of the frame for the use of fracture boards if necessary. Other beds have bed-rests combined with the frame, and there is also an admirably contrived dual-bottom bedstead. The "Asylum" bedstead is a good specimen of its class, and the requirements of epileptics are also well provided by bedsteads with swing-down sides and patent rivets. The catalogue also contains a very ingenious bed for use more especially in camping or travelling in hot countries. It combines a stretcher, a bed, and a mosquito tent. The bed is most convenient for transport, well made, and moderate in price. Simple frames for mosquito nets are also sold separately. Messrs. Chorlton provide a large variety of folding beds, arm-chair beds, bed-rests, leg-rests, and reclining and adjustable couches. A novelty in spring mattresses is introduced in the "Two-surface" mattress, which is reversible and gives a choice of elasticity. This mattress should prove a comfort to a bed-ridden patient, as the variety obtainable would help to relieve inevitable weariness. All the articles in the catalogue are illustrated and the list should certainly be procured for reference by those about to furnish privately or for a public institution.

The amount received in subscriptions during the past year by the committee of the Norfolk and Norwich Hospital shows an increase over last year of £115. The donations, too, for the past year showed a handsome increase of £944 odd, which is accounted for by the munificent gift of Sir R. P. Beauchamp, Bart., of £1,000. The income from investments also shows an increase.

The estimated outlay on the new model dwellings now being erected by the City of Westminster in Regency Street, Westminster, the foundation-stone of which was laid by the Prince of Wales the other day, is £95,000. It is estimated that the rents charged will pay a return of 3½ per cent. The buildings will occupy one and a half acres; they will contain 793 rooms, divided into 344 tenements.

